# PtpA protein from *Mycobacterium avium* subsp. *paratuberculosis* as a potential marker of rheumatoid arthritis in humans

**DOI:** 10.1371/journal.pone.0316727

**Published:** 2025-01-03

**Authors:** Jorge Hernández-Bello, Horacio Bach, Sergio Cerpa-Cruz, Gabriela Athziri Sánchez-Zuno, Rodolfo Hernández-Gutiérrez, Ferdinando Nicoletti, Andrea Saraceno, José Muñoz-Valle

**Affiliations:** 1 Instituto de Investigación en Ciencias Biomédicas, Centro Universitario de Ciencias de la Salud Universidad de Guadalajara, Guadalajara, Mexico; 2 Division of Infectious Diseases, Faculty of Medicine, The University of British Columbia, Vancouver, BC, Canada; 3 Division of Rheumatology, Guadalajara Civil Hospital "Fray Antonio Alcalde", Guadalajara, Jalisco, Mexico; 4 Department of Medicine, Yale School of Medicine, New Haven, CT, United States of America; 5 Biotecnología Médica y Farmacéutica, Centro de Investigación y Asistencia en Tecnología y Diseño del Estado de Jalisco, Guadalajara, Mexico; 6 Department of Biomedical and Biotechnological Sciences, University of Catania, Catania, Italy; Kyungpook National University, REPUBLIC OF KOREA

## Abstract

Studies have noted the connection between *Mycobacterium avium* subspecies *paratuberculosis* (MAP) and autoimmunity. MAP is an intracellular pathogen that infects and multiplies in macrophages. To overcome the hostile environment elicited by the macrophage, MAP secretes a battery of virulence factors to neutralize the toxic effects of the macrophage. One of the virulence factors is the Protein Tyrosine Phosphatase A (PtpA), a protein secreted by MAP that interferes in the phago-lysosome fusion, rendering the pathogen unnoticed in the cytoplasm of the macrophage. This study aimed to assess the presence of PtpA antibodies in the sera of Mexican individuals with rheumatoid arthritis (RA) and investigate its possible use as a biomarker for disease activity. We compared RA patients (n = 100) to control subjects (CS) (n = 100) by assessing specific immune responses to PtpA (the antigen) by an indirect ELISA method. Results showed a significant difference in PtpA levels between RA and CS, with RA patients having a median OD of 0.4645 compared to 0.1372 in CS. Antibodies against PtpA were present in 95% of RA patients and 16% of CS (AUC = 0.9163, p = 0.0001). Male control subjects showed higher PtpA reactivity than female CS. The Disease Activity Score (DAS-28) analysis showed that individuals with moderate to high disease activity had lower levels of PtpA reactivity. The results suggest a potential connection between RA and MAP infection.

## Introduction

Rheumatoid arthritis (RA) is a chronic, systemic, autoimmune disorder characterized by persistent inflammation and progressive joint damage, leading to significant disability and morbidity [1]. The exact cause of RA remains elusive; however, factors contributing to the development of RA can be broadly categorized into host-related (genetic predispositions, epigenetic changes, hormonal and reproductive aspects, and co-existing health conditions) and environmental influences (smoke and other airborne substances exposure, the presence and role of microbiota and infectious organisms, dietary habits, and socioeconomic considerations) [2].

*Mycobacterium avium* subsp. *paratuberculosis* (MAP), the etiological agent of Johne’s disease in ruminants, has been associated with human autoimmune diseases, such as Hashimoto’s thyroiditis, multiple sclerosis, Type 1 diabetes, Crohn’s disease, and rheumatoid arthritis [3–10]. MAP infects and multiplies within host macrophages, enabling it to persist in the host and potentially trigger chronic inflammatory responses [11]. MAP is a potential zoonotic pathogen that has been identified in a variety of animal species in Mexico, including wild ruminants in zoos, white-tailed deer in Coahuila, sheep in the hot-arid region of Sonora or the reproductive tissues and semen of naturally infected rams [12–15]. MAP is a recognized pathogen resistant to pasteurization, and it has been found in dairy products and infant formula [16–18]. These findings underline the potential for widespread exposure to MAP among humans through various routes, such as contaminated food, water, or direct contact with infected animals.

Among the proteins secreted by MAP, Protein Tyrosine Phosphatase A (PtpA) has been identified as a crucial player in the pathogenesis of MAP-related diseases [19, 20]. This protein is secreted during infection; therefore, it may play a role in interacting with host cell substrates and disrupting the processes required to eliminate internalized bacteria by immune cells. PtpA functions by dephosphorylating the host protein Vacuolar Protein Sorting 33B, which is involved in the fusion of the MAP-containing phagosome with the lysosome [21]. It has been suggested that this disruption might be partially related to the ability of MAP to endure within the macrophage [20]. This disruption is important in the RA context, as macrophages produce cytokines that promote inflammation and contribute to RA’s breakdown of bone and cartilage [22].

MAP proteins may trigger an immune response in the host; therefore, in a genetically susceptible individual, this immune reaction could potentially trigger an autoimmune response, thereby contributing to the development or progression of autoimmune diseases [7, 23]. However, the significance of these proteins in the pathogenesis and activity of RA remains to be fully elucidated.

In this study, we aim to assess the recognition of the PtpA protein secreted by MAP in the sera of Mexican patients with RA and explore its potential link with disease activity. We aim to assess the PtpA protein secreted by MAP as a marker for the diagnosis or disease activity of RA patients.

## Material and methods

### Subjects

RA patients (23 males, 77 females; median age 58) who met the 2010 ACR/EULAR Classification Criteria for RA (American College of Rheumatology) were enrolled and recruited at the Rheumatology Unit at the Civil Hospital of Guadalajara, Fray Antonio Alcalde, Guadalajara, Jalisco, Mexico. The sampling period extended from January 1, 2018, to December 31, 2021.

Data were gathered on various parameters, including the length of disease presence, the presence of rheumatoid factor (RF) and anti-cyclic citrullinated peptide (anti-CCP), the usage of steroid treatments and disease-modifying anti-rheumatic drugs (DMARDs), levels of the inflammation marker C-reactive protein (CRP), erythrocyte sedimentation rate (ESR) levels as an indicator of inflammation, the Disease Activity Score-28 (DAS-28) as a measure of disease activity, and the Health Assessment Questionnaire (HAQ) to evaluate patient health status [24, 25].

One hundred healthy control subjects (CS) (20 males, 80 females; median age 40 years) were also recruited in the same Hospital. The recruited subjects confirmed verbally that they did not have past tuberculosis infection.

### Ethics approval statement

This study received approval (No. 0122017) from the University of Guadalajara’s ethics committee and complied with the ethical guidelines in the Declaration of Helsinki (64th General Assembly, Fortaleza, Brazil, October 2013). Every participant provided written informed consent.

### ELISA

#### Plate preparation

Recombinant PtpA proteins were produced following an earlier protocol [20]. Briefly, PtpA was expressed in *Mycobacterium smegmatis*, containing the *ptpA* gene cloned into the hygromycin-resistant pALACE vector. Then, it was purified via affinity chromatography using Ni-NTA resin and stored at -20°C until required.

Maxisorp ELISA plates (TermoFisher) were prepared with 50 μg/mL of each antigen in phosphate-buffered saline (PBS) and left overnight at 4°C. On the following day, plates were rinsed three times with PBS supplemented with Tween-20 (PBS-T) and blocked with 3% bovine serum albumin (BSA) in PBS overnight at 4°C. The quantity of antigen employed in this study had been previously established as the required amount to achieve a noticeable alteration in the reading [19, 26, 27]. The following day, the blocking solution was removed, and the plates were air-dried at ambient temperature.

#### Assay procedure

The research used ELISA to scrutinize specific immune reactions to PtpA protein in patients with RA, compared to healthy individuals (CS). The procedure was conducted as detailed in [28]. Optical density (OD) measurements were taken at a 450 nm wavelength using an Epoch (BioTek, Whitney, US) microplate reader.

All tests were conducted at least three times, and all serum specimens were analyzed in duplicate. The base level, defined by the protein’s reaction to only a secondary antibody, was deducted from each result. Every experiment included positive control samples. The positivity threshold for each assay was computed through ROC analysis and set to achieve over 90% specificity, with sensitivity adjusted accordingly.

### Statistical analysis

The significance of discrepancies in OD readings between RA and CS was assessed using a Mann-Whitney test. Linear regression assessments were utilized to ascertain the relationship between RA characteristics and serum levels of antibodies against PtpA. Analysis of the area under the receiver operating characteristic curve (AU ROC) was accomplished with GraphPad Prism 6.0 software (San Diego, CA, USA). The Corrplot R package was used for correlation analysis. Variations with a p-value < 0.05 were considered statistically significant.

## Results

The demographic details and clinical attributes of the RA patients and controls are outlined in Table 1. The RA patients had an average age of 54 years (IQR: 41–61 years), which was significantly older than the control subjects’ average age of 40 years (IQR: 31–53 years), p<0.0001.

**Table 1:** Clinical and demographic features of RA patients and CS.

	RA n = 100	CS n = 100	P-value
Age, years	54 (41–61)	40 (31–53)	<0.0001
Female sex, n (%)	81 (81%)	80 (80%)	>0.9
HAQ (0–3)	0.75 (0.25–1.25)	–	
DAS-28	3.2 (2.60–5.10)	–	
CRP, mg/dL	6 (2–12)	–	
ESR, mm/h	20 (12–44)	–	
Steroids use, n (%)	1 (1%)	–	
DMARDs use, n (%)	73 (73%)	–	
NSAIDs use, n (%)	36 (36%)	–	
Contraceptives use, n (%)	5 (5%)	–	
Calcium supplementation	18 (18%)	–	
Active smokers	17 (17%)	–	
Smoking time, years	1 (0–20)	–	
Exposure to wood smoke	52 (52%)	–	
Exposure time to wood smoke, years	8 (0–20)	–	

RA = Rheumatoid Arthritis; CS = Control Subjects; HAQ = Health Assessment Questionnaire; DAS-28 = Disease Activity Score in 28 joints; CRP = C-reactive Protein; ESR = Erythrocyte Sedimentation Rate; DMARDs = Disease-Modifying Antirheumatic Drugs; NSAIDs = Nonsteroidal Anti-Inflammatory Drugs. Antirheumatic Drugs; NSAIDs = Nonsteroidal Anti-Inflammatory Drugs.

The distribution of genders was similar in both groups (>0.9), with women comprising 81% of the RA group and 80% of the control group.

RA patients had a median Health Assessment Questionnaire (HAQ) score of 0.75 (IQR: 0.25–1.25) and a median Disease Activity Score in 28 joints (DAS-28) of 3.2 (IQR: 2.60–5.10). This suggests that, on average, RA patients had mild to moderate impairment in their daily activities and functional abilities and moderate disease activity.

Regarding medication use, only 1% of RA patients were on steroids, while 73% were taking disease-modifying anti-rheumatic drugs (DMARDs). Nonsteroidal anti-inflammatory drugs (NSAIDs) were used by 36% of the patients.

**Fig 1:**
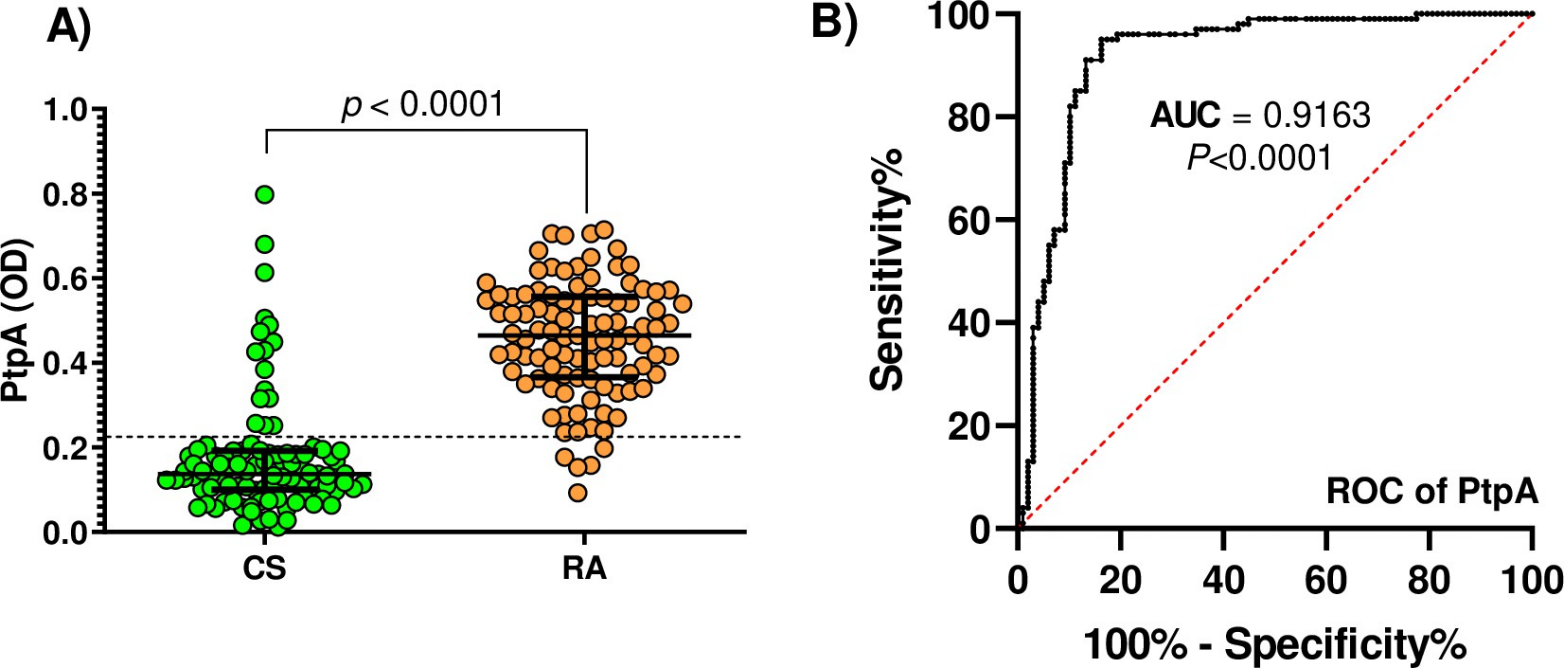
Serum antibody reactivity PtpA in RA subject and CS. Part A shows a comparison by study groups. Bars denote the median along with the interquartile range. Dashed lines indicate the thresholds for antibody positivity. P-values are shown above the distributions. Part B presents the ROC analysis for the PtpA protein.

Significant differences were observed when comparing PtpA levels between individuals with RA and CS (Fig 1A). The median OD PtpA level in RA was 0.4645 (IQR: 0.3660–0.5560), while in CS, it was 0.1372 (IQR: 0.1005–0.1917). Antibodies against PtpA were detected at levels above the positive threshold in 95% (95 out of 100) of the RA patients, compared to 16% (16 out of 100) of the CS. The area under the curve (AUC) in the receiver operating characteristic analysis was 0.9163 (p = 0.0001, Fig 1A and 1B).

**Fig 2:**
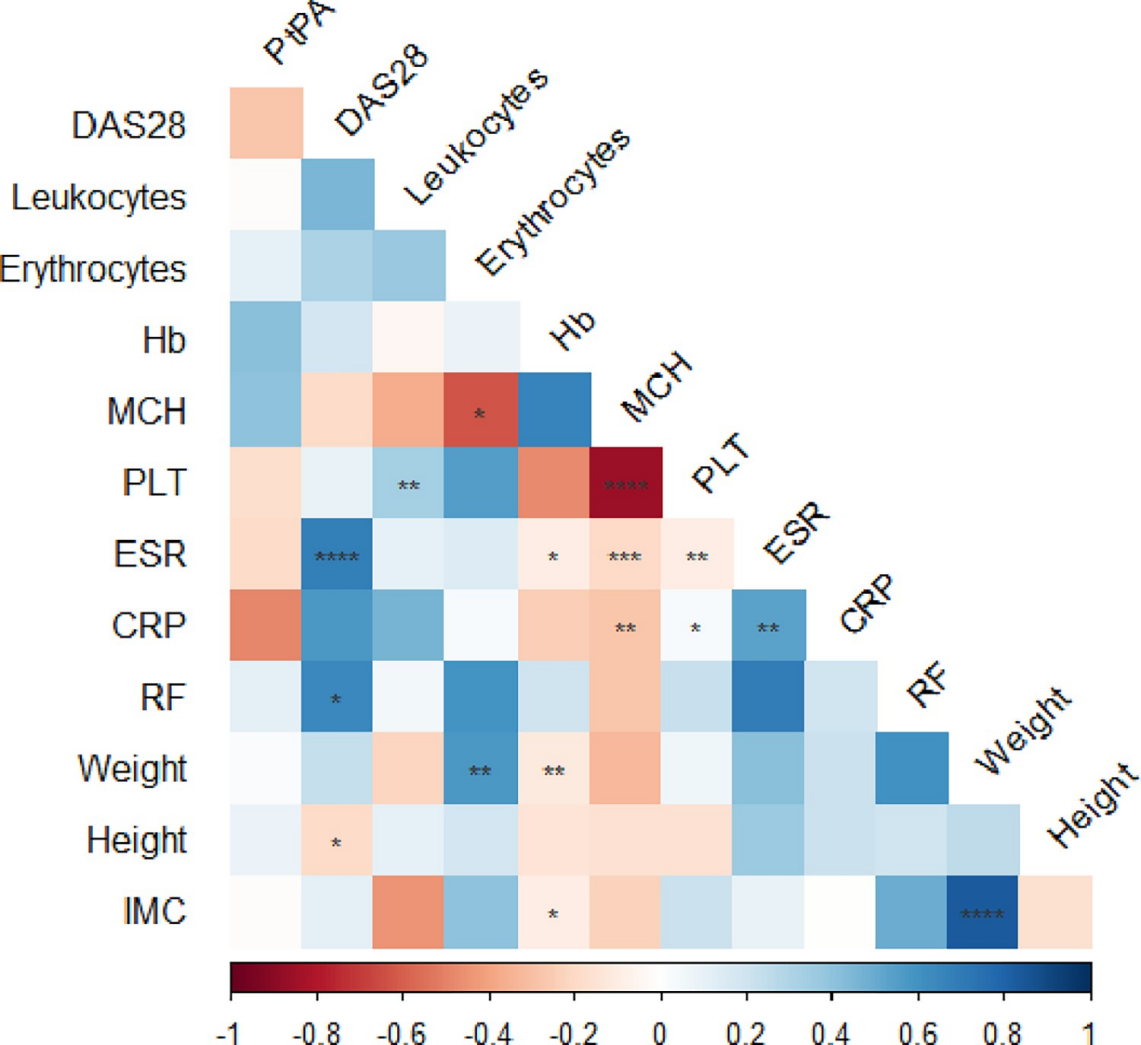
Correlation matrix of clinical features with PtPA reactivity in RA patients. The heat map summarizes Spearman´s correlation analysis results among the data considered. Correlation coefficients (r) are shown in the relative boxes between the considered variables at the intersection. Positive correlation coefficients are shown in the shadows of blue, while negative correlations are in the shadows of red. *, p<0.05; **, p<0.01; ***, p < 0.001; ****, p <0.0001.

A correlation matrix analysis found no statistically significant correlations between PtpA reactivity and clinical features in RA patients (Fig 2).

**Fig 3:**
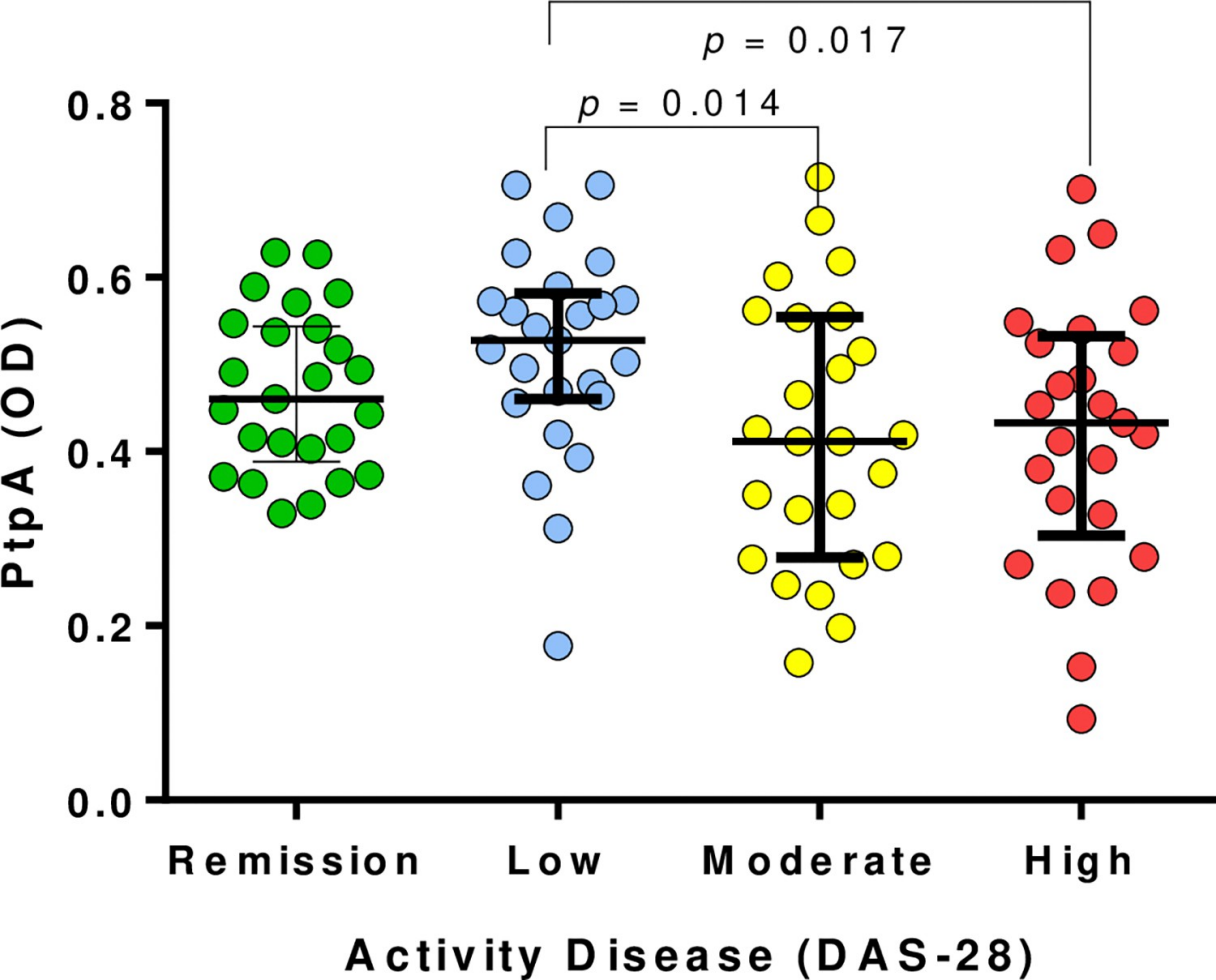
Association of serum antibody reactivity PtpA and DAS-28 score. A) Serum antibody reactivity PtpA in RA patients by Disease Activity Score (DAS-28); Bars represent the median ± interquartile range. *P*-values are indicated above the distributions.

However, the analysis of serum antibody reactivity to PtpA in RA patients, based on the DAS-28 score, revealed notable differences when categorizing the groups according to disease activity (remission, low, moderate, or high). A consistent trend was observed, indicating lower levels of PtpA reactivity as disease activity increased (Fig 3A). The median reactivity levels (OD) for remission, low, moderate, and high disease activity were 0.4600, 0.5277, 0.4120, and 0.4330, respectively. Significant differences were explicitly identified when comparing the groups with low and moderate disease activity (p = 0.014) and those with low and high disease activity (p = 0.017). However, the correlation analysis between serum antibody reactivity to PtpA (OD) and the DAS-28 score did not show statistical significance (Fig 3B).

**Fig 4:**
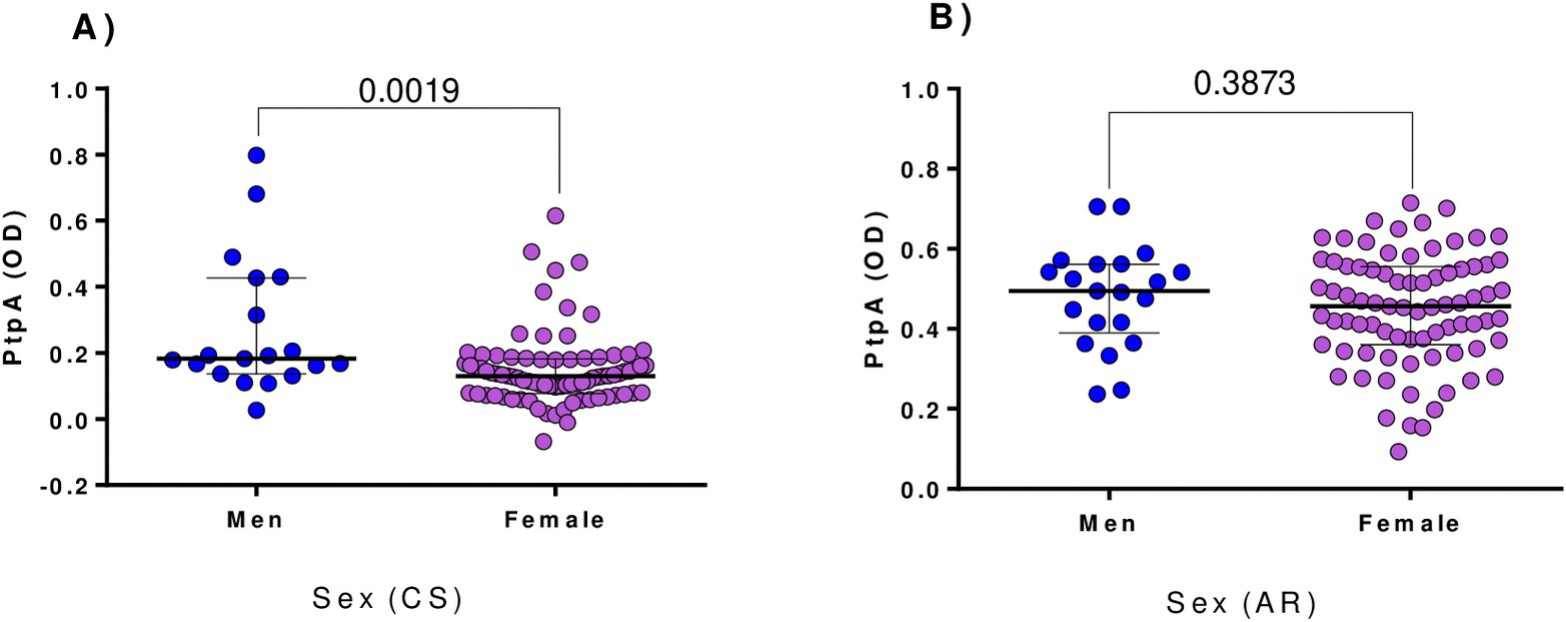
Serum antibody reactivity PtpA in RA subject and CS by sex. A) Comparison between CS; B) Comparison between RA patients. Bars represent the median ± interquartile range. *P*-values are indicated above the distributions.

When serum antibody reactivity to PtpA in RA and CS was compared by sex, significant differences were observed in CS; male subjects had higher reactivity levels than female subjects (OD = 0.18 vs 0.12, respectively). However, in the RA group, no significant differences in reactivity were found between sexes (OD = 0.49 vs 0.45; Fig 4B).

## Discussion

Several epidemiological and experimental studies suggest that bacterial infections might not only trigger the onset of RA but also influence its course and outcome [29]. Indeed, specific bacterial species, such as *Porphyromonas gingivalis*, *Proteus mirabilis*, *Mycobacterium* ssp., *Aggregatibacter actinomycetemcomitans*, and *Prevotella copri* have been linked to RA [30–32]. The association between these bacteria and RA is suggested to be due to molecular mimicry, wherein bacterial antigens resemble self-antigens, leading to an autoimmune response [31].

MAP is another bacterium implicated in RA, with evidence suggesting that MAP-derived proteins can stimulate an immune response that may contribute to RA pathogenesis [28]. Notably, molecular mimicry also may play a pivotal role in this process. In recent studies, homologous BOLF1 (an Epstein-Barr virus protein) and MAP epitopes are significantly correlated with the Interferon Regulatory Factor 5 (a critical regulator of the immune response). Therefore, it has been suggested that exposure to MAP may trigger an immune response against self-proteins similar to MAP epitopes, which may lead to RA onset or progression [28, 33]. Also, MAP is the only pathogenic mycobacteria that enhances the secretion of tumor necrosis factor-alpha (TNF-α) [34], a known proinflammatory cytokine secreted by infected macrophages.

The present study supports the possible role of PtpA and MAP in the pathogenesis of RA. Results showed that 95% of RA patients had antibodies against PtpA above the threshold, compared to only 16% of CS (AUC of 0.9163). These findings support previous research that identified PtpA as an immunogenic agent for MAP-related diseases, such as arthritis, multiple sclerosis, Hashimoto’s thyroiditis, Crohn’s disease, systemic lupus erythematosus, and type 1 diabetes mellitus [4, 7, 8, 11, 27, 35]. Based on the AUC value, antibodies against PtpA could be a robust biomarker for RA. Although these results are promising, more studies are needed to verify them in larger populations and evaluate anti-PtpA antibodies’ potential in RA diagnosis or prognosis.

Despite the pronounced antibody response against PtpA in RA patients, we did not find significant correlations between PtpA reactivity and clinical features in RA patients. However, we observed a consistent trend indicating lower levels of PtpA reactivity in those individuals with moderate or high disease activity. This observation may reflect the intricate relationship between immune response, disease activity, and the role of MAP in RA. It hints at a scenario where a heightened immune response against PtpA might be implicated in the earlier stages of the disease, subsequently decreasing as the disease progresses. An alternative interpretation of our observations is that the infection of macrophages by MAP and the subsequent secretion of PtpA could impact macrophage phenotypes and modulate the immune response in a manner that varies with the level of clinical activity in RA.

Macrophages are highly versatile immune cells critical in initiating and resolving inflammation. Their function is known to be affected in RA [22]. The interaction of macrophages with MAP and its secreted proteins, such as PtpA, could lead to alterations in macrophage polarization, inducing differential immune responses [20].

It is also plausible to consider the role of different treatment regimens in this phenomenon. Treating patients with moderate or high disease activity is typically more potent and may impact the immune response to MAP and antibodies against PtpA. This hypothesis aligns with a previous study on CD that found different drug treatments affected the levels of antibodies against PtpA because treatment could impact MAP survival and, consequently, CD progression [27]. Meanwhile, previous studies have suggested that MAP infection could be a factor in the development of RA [33]. Therefore, further research is needed to fully understand the complex relationship between RA treatments, MAP survival, and the immune response to MAP.

One limitation of the present study is that it included a relatively small number of patients, which may restrict the generalizability of our results and limit the detection of subtler associations or effects. Furthermore, most of our patients were being treated with multiple drugs, adding additional complexity to the interpretation of our results. Given the possible influence of treatment on MAP survival and, consequently, on the immune response to MAP, future studies should aim to evaluate this effect in patients on monotherapies. Future studies addressing the limitations could provide a more nuanced understanding of the role of PtpA in RA and its potential as a diagnostic or prognostic biomarker.

Along this line of research, evaluating whether reactivity to PtpA occurs in individuals at risk for developing RA and whether it represents a predictive biomarker for disease development will be interesting.

Finally, our analysis revealed a sex-based difference in PtpA reactivity within the CS group, with male subjects showing higher reactivity than female subjects. This finding aligns with the known sex-based differences in immune responses and susceptibility to autoimmune diseases [36]. However, this pattern was not observed in the RA group, which might be due to the overarching influence of the disease overshadowing any potential sex-based differences in the immune response.

## Conclusion

Comprehensive data on the prevalence of MAP in the human population in Mexico are currently lacking. Given the zoonotic potential of MAP and its suggested links to several human diseases [3], there is a clear need for seroprevalence studies in the Mexican population to assess the extent of MAP exposure and its potential implications for human health.

Our findings add to the growing body of evidence highlighting the potential role of MAP, specifically PtpA protein, in the context of RA. Further research is needed to clarify these findings’ implications and explore the potential utility of PtpA as a diagnostic or a biomarker for RA.

## Supporting information

S1 Data (XLSX)
